# Xerostomia, Hyposalivation, and Salivary Flow in Diabetes Patients

**DOI:** 10.1155/2016/4372852

**Published:** 2016-07-10

**Authors:** Rosa María López-Pintor, Elisabeth Casañas, José González-Serrano, Julia Serrano, Lucía Ramírez, Lorenzo de Arriba, Gonzalo Hernández

**Affiliations:** Department of Oral Medicine and Surgery, School of Dentistry, Complutense University, 28040 Madrid, Spain

## Abstract

The presence of xerostomia and hyposalivation is frequent among diabetes mellitus (DM) patients. It is not clear if the presence of xerostomia and hyposalivation is greater in DM than non-DM patients. The aims of this systematic review are (1) to compare the prevalence rates of xerostomia, (2) to evaluate the salivary flow rate, and (3) to compare the prevalence rates of hyposalivation in DM versus non-DM population. This systematic review was conducted according to the PRISMA group guidelines by performing systematic literature searches in biomedical databases from 1970 until January 18th, 2016. All studies showed higher prevalence of xerostomia in DM patients in relation to non-DM population, 12.5%–53.5% versus 0–30%. Studies that analyzed the quantity of saliva in DM population in relation to non-DM patients reported higher flow rates in non-DM than in DM patients. The variation flow rate among different studies in each group (DM/CG) is very large. Only one existing study showed higher hyposalivation prevalence in DM than non-DM patients (45% versus 2.5%). In addition, quality assessment showed the low quality of the existing studies. We recommend new studies that use more precise and current definitions concerning the determination and diagnosis of DM patients and salivary flow collection.

## 1. Introduction

Diabetes mellitus (DM) is an endocrine disease characterized by a deficit in the production of insulin with consequent alteration of the process of assimilation, metabolism, and balance of blood glucose concentration. DM has become a worldwide public health problem. In recent years, the global prevalence of DM has increased substantially, reaching 8.3% in 2014, which corresponds to 387 million patients [1]. Essentially, there are two types of DM: type 1 DM (T1DM) and type 2 DM (T2DM). T1DM accounts for approximately 5% of diagnosed diabetes cases [2].

Xerostomia is a subjective complaint of dry mouth, whereas hyposalivation is an objective decreased of salivary flow. The clinical method most often employed for the diagnosis of salivary dysfunction is a sialometry test. Hyposalivation is considered to appear when salivary flow rates are under 0.1 mL/min at rest (UWS) or 0.7 mL/min under stimulation (SWS). Xerostomia is often associated with hyposalivation, but not always. And many cases of xerostomia have been described in patients with a normal salivary flow rate [3–6].

Several factors are capable of inducing salivary disorders in DM patients such as ageing, head and neck radiotherapy, systemic disorders, and several drugs [5]. Systemic diseases associated with xerostomia include rheumatologic chronic inflammatory disorders (Sjögren syndrome, rheumatoid arthritis, and systemic lupus erythematosus), endocrine disorders (DM, hyperthyroidism, and hypothyroidism), neurologic disorders (depression and Parkinson's disease), genetic disorders, metabolic disorders (dehydration, bulimia, anaemia, and alcohol abuse), infectious disorders (HIV/AIDS, HCV infection), and others (fibromyalgia, graft-versus-host-disease, sarcoidosis, and chronic pancreatitis). Many cases of xerostomia are also related to psychological conditions like depression and anxiety [5, 6].

Both types of DM, T1DM and T2DM, have been associated previously with xerostomia [7–12]. There are also studies that have showed a decreased salivary flow in DM patients in relation to non-DM patients [7, 8, 12–21]. The reason for these problems could be due to damage to the gland parenchyma, alterations in the microcirculation to the salivary glands, dehydration, and disturbances in glycemic control [5].

Considerable debate exists surrounding the issue, if the presence of xerostomia and hyposalivation is greater in DM than non-DM patients. No systematic review has been performed up to now. Given the lack of systematic knowledge, we have conducted the first systematic review concerning the prevalence of xerostomia and hyposalivation in DM (compared to non-DM) patients. We also have analyzed the differences in the rate of salivary flow between DM and non-DM patients.

The main objectives of this review were (1) to compare the prevalence rates of xerostomia in the DM and non-DM population, (2) to evaluate the salivary flow rate in the DM and non-DM population, and (3) to compare the prevalence rates of hyposalivation in the DM and non-DM population.

## 2. Materials and Methods

The systematic review was performed according to the PRISMA (Preferred Reporting Items for Systematic Reviews and Meta-Analyses) guidelines [23].

### 2.1. Focused Question

Based on the PRISMA guidelines, 3 focused questions were constructed. The addressed focused questions (PICO) were as follows: (1) Do DM patients have higher xerostomia prevalence than non-DM patients? (2) Is the salivary flow rate lower in DM patients compared to non-DM patients? (3) Do DM patients have higher hyposalivation prevalence than non-DM patients?

### 2.2. Search Strategy

A comprehensive literature search was conducted by searching the international biomedical literature databases. PubMed/MEDLINE (National Library of Medicine, Bethesda, Maryland), Scopus, and Cochrane database were searched from 1970 until January 18th, 2016, using different combinations of the following keywords: diabetes; xerostomia; dry mouth; hyposalivation; and salivary flow. Moreover, we performed an additional handsearch to find potential eligible studies as reference lists of review articles and relevant studies.

### 2.3. Study Selection

#### 2.3.1. Inclusion Criteria

Full-text articles were included if they met the inclusion criteria with respect to types of studies, types of population, and the main outcome/s regardless of the time period of study and the year of publication.

*Types of Studies*. The studies had to be (1) original studies, (2) cross-sectional studies, (3) comparative studies (DM group and healthy control group (CG)), and (4) only in humans. As we evaluated prevalence rates review articles, experimental studies, longitudinal studies, case-reports, commentaries, and Letters to the Editor were excluded. We did not include unpublished articles.

*Types of Population*. Individuals with diabetes could have T1DM or T2DM. We also considered other diabetes classifications, namely, insulin-dependent (IDDM) and non-insulin-dependent DM (NIDDM). The total population with DM did not have to suffer specific diseases apart from DM (e.g., end-stage renal disease and hypertension). Individuals without DM were also considered with the aim of comparing prevalence and flow rates between the DM and non-DM population. Individuals without DM did not have to have specific diseases.

*Outcomes*. The definitions of xerostomia, quantity of salivary flow rate, and hyposalivation are detailed below. Different questions to assess xerostomia were considered: Does your mouth feel dry frequently? Does your mouth usually feel dry, especially during meals? Does your mouth feel dry when you are eating a meal? Do you have difficulties swallowing foods if you eat without additional fluids? Positive response to one of these questions and the consideration of patient's subjective feeling of dry mouth were considered to be xerostomia. Different types of salivary flow rate were considered: UWS (nonstimulated salivary flow), SWS (stimulated salivary flow), USP (nonstimulated parotid flow), SSP (stimulated parotid flow), and SSS (stimulated submandibular/sublingual flow). Furthermore, hyposalivation was considered when UWS < 0.1 mL/min or SWS < 0.7 mL/min, but we included studies that considered hyposalivation when UWS < 0.3 mL/min and SWS < 0.5 mL/min. The main outcomes were the prevalence of xerostomia and/or hyposalivation in percentage and/or the quantity of salivary flow rate in mL/min.

#### 2.3.2. Exclusion Criteria

Studies were excluded if they were published in a language other than English. They were also excluded if they solely reported prevalence of xerostomia/hyposalivation and salivary flow rates among persons with DM in relation to the total population (DM and non-DM) and not exclusively to the diabetic (possibly compared to the non-DM) population.

### 2.4. Data Collection and Extraction

Two authors (Rosa María López-Pintor and Elisabeth Casañas) independently screened all the retrieved titles and abstracts identified through the search strategies to identify potentially eligible articles. Full texts of relevant studies judged by title and abstract were read and independently assessed with reference to the eligibility criteria by two authors (Rosa María López-Pintor and José González-Serrano). Disagreements were resolved by discussion with a third reviewer (Julia Serrano). Data extraction was performed including information about first author, publication year, country, study population, mean age, type of DM, DM diagnosis (if available), definition of xerostomia, definition of hyposalivation (if available), type of flow rate, and data sources of the study. With regard to the results, xerostomia prevalence (%) and salivary flow rate (mL/min), as well as hyposalivation prevalence (%) of DM and non-DM groups, were extracted. The reported statistical signification was extracted if it was available.

### 2.5. Quality Assessment

In the final selection of eligible studies, we assessed features that could potentially bias the estimates of xerostomia/flow rate/hyposalivation using the Joanna Briggs Institute Prevalence Critical Appraisal Tool (Table 1) [24]. Using this tool we defined criteria based on clinical and epidemiological expertise and ranked potential sources of bias into low or high risk of bias. Scores of 0–5 were evaluated as “low quality” while those of 5–10 were considered to indicate “high quality.”

Critical appraisal was conducted by two reviewers (Gonzalo Hernández and Lucía Ramírez) independently of each other. The reviewers met to discuss the results of their critical appraisal; if the two reviewers disagreed on the final critical appraisal and could not be resolved through discussion, a third reviewer (Julia Serrano) was required.

### 2.6. Categorization of Studies

Due to the high heterogeneity of the studies, we analyzed the outcomes of interest in accordance with the prevalence of xerostomia or salivary quantity flow rate/hyposalivation (if available), type of DM, and age (adults ≥ 19 years old/children and adolescents). There were studies that reported xerostomia prevalence and flow rate; therefore, there could be two groups. The following categories were the result: (1) xerostomia studies in adults T2DM, (2) xerostomia studies in adults NIDDM, (3) xerostomia studies in children and adolescents T1DM, (4) salivary flow rate studies in adults T1DM, (5) salivary flow rate studies in adults IDDM, (6) salivary flow rate/hyposalivation prevalence studies in adults T2DM, (7) salivary flow rate/hyposalivation prevalence studies in children and adolescents T1DM, and (8) salivary flow rate/hyposalivation prevalence studies in children and adolescents IDDM.

### 2.7. Statistic Methods

The results of xerostomia prevalence from the included studies were presented as a percentage. The results of quantity salivary flow rate were presented as mean ± standard deviation (if available). Hyposalivation prevalence results were shown as a percentage. The age of different populations was presented as mean ± standard deviation, but there were studies that categorized the age or presented only the mean. We showed the possible statistical signification if it was available.

Due to heterogeneity of results, we did not perform a meta-analysis.

## 3. Results

### 3.1. Searching and Inclusion

**Figure 1:**
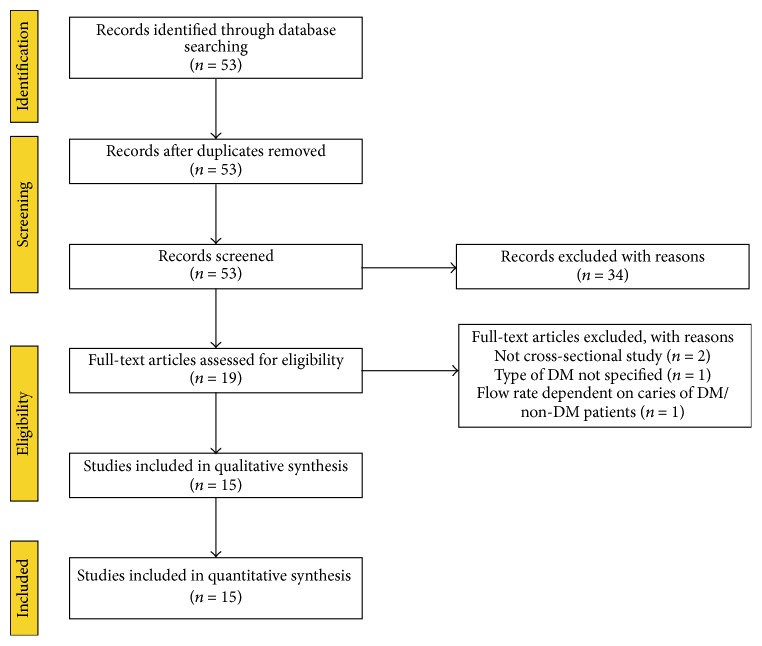
Flowchart of the systematic review process.

The initial search yielded 53 studies. Thirty-eight studies, which did not fulfill the eligibility criteria, were excluded (the Appendix). A total of 15 articles were included and processed for data extraction. The selection procedure is presented in Figure 1.

### 3.2. Study Design and Quality Assessment

With regard to the main outcome, 7 papers considered xerostomia prevalence (Table 2), and 12 articles considered quantity of salivary flow rate in DM patients (Table 3), while 4 papers considered both. Only one paper about salivary flow rate in DM population considered hyposalivation prevalence as outcome (Table 3). The results are presented in two parts, xerostomia studies and salivary flow rate/hyposalivation studies.

#### 3.2.1. Xerostomia Studies

We found 7 studies about xerostomia prevalence that met our inclusion criteria. Two of them, written by Sandberg et al. [9, 10], presented the same study population. Therefore, we considered these two studies as one study in Table 2. The majority of studies that reported prevalence of xerostomia in DM patients were performed in adults (*n* = 6), 5 studies in T2DM patients and one in NIDDM. Only one study was performed in children and adolescents T1DM. One study carried out in adults T2DM [18] did not show xerostomia prevalence rates, but it was included due to presence in the results of explanation of no significant correlation in xerostomia in DM/CG patients.

With respect to the recruitment of patients, three studies had selected their DM patients from an endocrinology service or a diabetic care unit of a specialized medical care or hospital, two from a geriatric center and one (the two studies realized by Sanberg et al. [9, 10] with the same population) had sourced the DM patients from a register of primary health care. Control patients were selected from oral health centers (*n* = 4) and geriatric centers (*n* = 2). The studies included a minimum of 29 and a maximum of 102 DM patients and 18–102 control patients. Only two studies specified the DM diagnosis, one WHO criteria 2006 (fasting blood glucose greater ≥ 126 mg/dL) and another one blood glucose levels ≥ 140 mg/dL at 2 hours after oral glucose tolerance test. No one study reported duration of DM and three studies [8, 12, 18] reported the HbA1_c_ levels and classified the patients in well controlled DM (WCDM) and poorly controlled DM (PCDM).

DM and CG participants were matched by gender in 4 studies, by age in 5 studies, by race distribution in one, by diuretics and antidepressants treatment in one, and by socioeconomic status in another one. With regard to statistical significance, three studies [8–10, 12] found that DM patients had more significant xerostomia prevalence than non-DM patients. Only one study [18] did not realize the appropriate statistical methods.

Regarding quality assessment all studies obtained scores ≤5; therefore the studies were evaluated as “low quality” (Table 2). Due to the poor quality of the included studies no meta-analysis was performed.

#### 3.2.2. Salivary Flow Rate/Hyposalivation Studies

We found 12 studies about quantity of salivary flow rate that met our inclusion criteria; one of them considered hyposalivation prevalence as outcome (Table 3). The majority of studies were carried out in adults (*n* = 8), 6 studies in T2DM patients, one in T1DM patients, and another one in IDDM. Four studies were carried out in children and adolescents, 2 in T1DM patients and 2 in IDDM.

Three studies recruited their DM patients from a diabetes care unit of a hospital, 3 from an endocrine unit, 3 from a pediatric endocrinology service, one from a university dental school, one from an oral health study, and another one from community-living/geriatric centers. Non-DM patients came from varied origins: oral health centers (*n* = 3), Swedish register (*n* = 1), healthy volunteers from a hospital staff (*n* = 1), members of a university community (*n* = 1), patients of a university dental school (*n* = 2), and participants in an oral health study of aging (*n* = 1), and 3 studies did not specify the origin. The studies included a minimum of 10 and a maximum of 243 DM patients and a minimum of 10 and a maximum of 240 non-DM patients.

Five studies specified the DM diagnosis, two WHO criteria 2006 (fasting blood glucose ≥ 126 mg/dL), one modified WHO criteria 2006 (fasting blood glucose ≥ 126 mg/dL) or currently taking diabetic medications, one blood glucose levels ≥ 140 mg/dL at 2 hours after oral glucose tolerance test, and the last one American Diabetes Association criteria 2010 (HbA1_c_ levels ≥ 6.5% or fasting blood glucose ≥ 126 mg/dL). One study [13] reported that DM patients suffered T1DM since childhood, and there was another study [21] that only included newly diagnosed diabetic children. With respect to dental condition, one study [7] did not include edentulous patients, one study [16] recruited only patients wearing complete maxillary or maxillary and mandibular dentures, and another one [8] excluded patients using total prostheses and mouth breathers. Four studies [8, 12, 13, 18] reported the HbA1_c_ levels and classified the patients in WCDM and PCDM.

DM and non-DM participants were matched by gender in 7 studies, by age in 6 studies, by race distribution in 2, by socioeconomic status in 3, by living in the same area in two, and by Tanner puberty states in another one. With regard to the type of flow rate 9 studies collected UWS, 4 SWS, 2 USP, one SSS, one USS, and one collected SSP.

Three studies did not explain the hour of collection of saliva and 4 studies did not specify the saliva collection duration. Two studies collected salivary flow during 10 minutes and 6 studies during 5 minutes. Five studies [13, 17, 18, 20, 21] did not show or clarify correctly the statistical methods. Regarding quality assessment, only one study [13] obtained JBI scores ≥5 (Table 3). Therefore, due to the poor quality of the majority of the included studies no meta-analysis was performed.

Only one study reflected prevalence of hyposalivation as outcome [7]. The definition of hyposalivation was UWS < 0.1 mL/min and SWS < 0.5 mL/min (actually <0.7 mL/min is considered). The study showed that DM patients had significantly greater hyposalivation prevalence than CG.

### 3.3. Main Findings

#### 3.3.1. Prevalence of Xerostomia in the DM/CG Population

The prevalence of xerostomia was analyzed in 7 studies (Table 2). In adults T2DM xerostomia prevalence varied between 12.5% and 53.5%, compared to 0–28.4% in the CG [7–10]. Only three studies [8–10] (two with the same study population [9, 10]) showed that DM patients suffered significantly more xerostomia than non-DM patients. One study realized by Bernardi et al. [8] showed that PCDM patients suffered more xerostomia prevalence than WCDM patients, 54% and 47%, respectively.

There was only one study about xerostomia in adults NIDDM [11]. This study showed that prevalence of xerostomia in NIDDM patients is greater than in CG population, 50% versus 30%, but this result was not significant.

Only one work was realized in children and adolescents T1DM between 10 and 19 years old. This study showed that prevalence of xerostomia was greater in T1DM patients than non-T1DM patients (0%), and the prevalence was greater in PCDM patients (100%) than WCDM patients (80%).

#### 3.3.2. Quantity of Salivary Flow Rate in the DM/CG Population

The quantity of salivary flow rate was analyzed in 12 studies (Table 3). There was only one study in adults T1DM [13]; this study showed that SWS flow rate was lower in DM versus non-DM patients, 1.30 versus 1.54 mL/min, and obtained higher salivary flow rate in PCDM than WCDM (1.31 versus 1.34 mL/min). The study did not show significant statistical results. In adults IDDM it was another study [22] that found significantly lower UWS flow rate in DM patients than non-DM patients, 0.35 ± 0.24 versus 0.48 ± 0.23 mL/min.

A considerable part of studies were realized in adults T2DM [7, 8, 15–18]. Four of them evaluated UWS [7, 15, 17, 18]; the UWS flow rate in T2DM and non-T2DM patients varied between 0.16–0.5 mL/min and 0.26–0.75 mL/min, respectively. Two of these studies [7, 15] obtained greater significant UWS flow rate in T2DM than in CG patients. In addition, Chavez et al. [18] assessed the UWS flow rate in WCDM and PCDM adults T2DM; they found higher rates in PCDM than WCDM.

Three studies assessed SWS flow rate in T2DM [7, 8, 16]. The rates of SWS in T2DM and non-T2DM patients varied between 0.63–0.95 mL/min and 1.14–1.95 mL/min, respectively. Two of them [7, 8] showed significant statistical results. The study of Bernardi et al. [8] showed that WCDM had greater SWS rates than PCDM.

USP flow rates were analyzed in two studies [17, 25]; only in one of them [17] did T2DM patients show lower rates than non-DM patients; none obtained significant results.

There were four studies [12, 19–21] that reported salivary flow rates in children and adolescents T1DM and IDDM between 4 and 19 years old. All studies evaluated UWS; the rates in DM population varied between 0.15 and 0.79 mL/min and in non-DM patients 0.25 and 1.06 mL/min. Three studies [12, 19, 20] obtained significant lower rates in T1DM and IDDM patients. Javed et al. [12] showed that WCDM had greater UWS rates than PCDM, but this result was nonsignificant.

#### 3.3.3. Prevalence of Hyposalivation in the DM/CG Population

Only one study evaluated this outcome and showed that hyposalivation prevalence was significantly greater in T2DM versus CG patients, 45% versus 2.5%.

## 4. Discussion

Multiple epidemiologic studies have suggested that xerostomia is frequent among DM patients. In addition, there are studies that have showed that DM patients presented lower salivary flow rates than non-DM population [26]. These salivary disorders could be associated with a poor quality of life and could increase the susceptibility to caries and oral infections in DM patients, particularly when there has been dehydration and inadequate blood glucose control [18]. DM is probably the most frequent metabolic disease with salivary implications, due to its high frequency. This systematic review was performed to analyze the prevalence of xerostomia and hyposalivation and the rates of salivary flow in DM patients in relation to non-DM patients. We specified explicit eligibility criteria, conducted comprehensive searches, and assessed risk of bias using criteria specific to this review.

### 4.1. Risk of Bias within Studies

Selection bias regarding the study population was minimized through the restriction to population-based studies. At the same time, we detected some sources of information bias. Firstly, the majority of studies [7, 9–13, 15, 16, 20–22] do not specify the DM diagnosis. Secondly, most of the studies [7, 8, 11, 12, 16, 18, 20–22] did not show the observation period and the type of recruitment of DM cases. With respect to the salivary flow rate, not all the studies reported the same type of salivary flow and the same technique, and these could also cause bias. Finally, DM and non-DM are not correctly matched; there are studies that did not even match age and gender [8, 12, 15, 19, 20] and there is no study that matched correctly the use of drugs and illness (apart DM), so important in xerostomia/hyposalivation etiology. As we can see in Tables 2 and 3, the sample size in the majority of studies was small (especially in adults T2DM), considering that DM is a very frequent disease. With respect to the statistical analysis, not all the studies reported continuous variables in mean ± standard deviation.

### 4.2. Risk of Bias across Studies

Due to the fact that only articles published in the English language were reviewed, publication (language) bias could not be ruled out. Although we searched three databases, we cannot guarantee that some related papers might not have been identified. However, we did check the reference lists of reviewed articles to identify relevant studies. The studies reviewed presented different types of DM and DM and non-DM patients of different age (see Section 2) that could cause detection bias. We minimized it by grouping together studies with similar age and the same DM type in every outcome.

### 4.3. Main Findings

We identified 15 studies reporting prevalence of xerostomia/hyposalivation and rates of salivary flow in DM population. Comparisons between studies were limited due to different types of DM, different types of salivary flow, and heterogeneous demographic characteristics (age, ethnic origin) of the studied individual. In addition, the quality assessment of studies was low. Hence, no quantitative data synthesis was performed. Nevertheless, there are some patterns that can be described.

#### 4.3.1. Xerostomia Prevalence

All studies about this outcome showed higher prevalence of xerostomia in DM patients in relation to non-DM population, 12.5%–53.5% compared to 0–30% [7–12, 18]. Nevertheless, only four studies [8–10, 12] (two with the same study population [9, 10]) have shown significant statistical results. Two studies [8, 12] showed that WCDM patients have lower xerostomia prevalence than PCDM.

#### 4.3.2. Salivary Flow Rates

All studies [7, 12, 15, 17–22] that analyzed the quantity of UWS in DM population in relation to non-DM patients reported higher UWS rates in non-DM than in DM patients. The variation flow rate among the different studies in each group (DM/CG) is very large. Six [7, 12, 15, 19, 20, 22] of these studies showed significant statistical results. The large variation flow rate among the studies could be due to the different criteria used to measure UWS. The time of measurement strongly influences the flow rate, so the saliva test (not only UWS) has to be performed at a fixed time-point of a limited time interval early morning due to the circadian rhythm of salivary flow [4, 27]. In addition, the duration of salivary collection is also important [4], and not all studies reflected the same duration. In the studies, where the time of flow rate collection is present, this time varied between 5 and 10 minutes. In addition, it is not clear if WCDM patients have higher UWS rates than PCDM; of two studies [12, 18] discussing this topic only one [12] showed nonsignificant higher rates for WCDM patients.

The comparison of the SWS rates between DM and non-DM patients showed that rates were higher in non-DM patients [7, 8, 13, 16], but only half of the studies showed significant statistical results [7, 8]. The SWS flow rate varies very much among the different studies, in the manner of UWS; the possible reason was specified previously.

#### 4.3.3. Hyposalivation Prevalence

Only one study [7] was about hyposalivation; this study showed significant statistical higher hyposalivation prevalence in DM than non-DM patients (45% versus 2.5%). The hyposalivation SWS level in this study is not actually accepted (<0.7 mL/min) if not <0.5 mL/min; therefore, the results could be biased.

### 4.4. Strengths and Limitations

The selection of studies for this systematic review was based on a systematic search approach with clearly determined search strategies. We included only those studies reporting xerostomia prevalence/salivary flow rate/hyposalivation within the DM population in relation to a non-DM control group. Moreover, we analyzed these outcomes in separate groups according to age and type of DM. This approach allows limited comparison of the studies despite a high degree of heterogeneity. Our review also has some limitations. Although three databases were searched, we cannot rule out having missed relevant studies, also due to publication bias. The studies published in languages other than English were not included. Most studies reporting our outcomes were conducted in economically developed areas such as USA and Sweden and thus do not represent a worldwide perspective.

In addition, there are studies previous to the year 2000. The change in the diagnostic criteria for DM from 140 mg/dL (7.8 mmol/L) to 126 mg/dL (7.0 mmol/L) in the fasting plasma glucose level in 1997 [28] led to an increase of the diabetic population due to the inclusion of less severe stages of the disease, and this must be taken into consideration when interpreting the results. Criteria for the diagnosis of prediabetes and DM could change periodically [2]; therefore, it is very important to realize the studies according to the current criteria.

## 5. Conclusions

The review conducted demonstrated the considerable variation in prevalence of xerostomia and salivary flow rates among DM population in relation to non-DM patients. Most studies found a higher prevalence of xerostomia and lower salivary flow rates in DM with respect to CG. We found only a study about hyposalivation that showed higher prevalence in DM than non-DM patients. A few studies showed that WCDM patients have lower xerostomia prevalence and higher salivary flow rates than PCDM patients. Owing to the high degree of heterogeneity regarding the types of DM, diagnosis of DM, age of patients, and types and techniques of salivary flow collection, it was difficult to compare the studies. In addition, the quality assessment showed the low quality of the existing studies. Therefore, the results of this systematic review were inconsistent.

We recommend that new studies analyzing the xerostomia and salivary flow rate in the DM population should use more precise and current definitions concerning the determination and diagnosis of DM patients and salivary flow rate collection. New studies should match correctly DM and non-DM patients, keeping in mind xerostomia associated drugs and illness (other than DM). New studies are required that consider hyposalivation in DM patients because a reduction in salivary flow is not always pathological.

## Figures and Tables

**Table 1:** JBI critical appraisal checklist for studies reporting prevalence data.

Assessment items	Yes	No	Unclear	Not applicable
(1) Was the sample representative of the target population?				
(2) Were study participants recruited in an appropriate way?				
(3) Was the sample size adequate?				
(4) Were the study subjects and the setting described in detail?				
(5) Was the data analysis conducted with sufficient coverage of the identified sample?				
(6) Were objective, standard criteria used for the measurement of the condition?				
(7) Was the condition measured reliably?				
(8) Was there appropriate statistical analysis?				
(9) Are all important confounding factors/subgroups/differences identified and accounted for?				
(10) Were subpopulations identified using objective criteria				

**Table 2:** Xerostomia prevalence studies.

Author, publication year, country	Study population (DM/CG)	Mean age (years) DM/CG	Type of diabetes	DM diagnosis	Definition of xerostomia	Xerostomia DM/CG%	Significant association	Matched variables (DM/CG)	JBI scoring
(1) *Studies in adults T2DM*									

Vasconcelos et al. 2010, Brazil [7]	40/40 (i) DM: endocrinology service of center for specialized medical care (ii) CG: Stomatology Clinic of School of Dentistry (iii) Smokers, drinkers, pregnant, edentulous, receptors of salivary gland surgery, radiotherapy of the head and neck region, Sjögren syndrome, rheumatoid arthritis, or lupus erythematosus excluded	57.7 ± 8.9/50.2 ± 12.3	T2DM	NS	Does your mouth feel dry frequently?	12.5%/2.5%	No	Gender Age	3

Bernardi et al. 2007, Brazil [8]	82/18 (i) DM: diabetic care unit of a local hospital (ii) CG: oral health center (same city) (iii) Those using total prostheses and mouth breathers were excluded. (iv) WCDM: HbA1_c_ ≤ 8% (23%) (v) PCDM: HbA1_c_ > 8% (77%)	PC 54.3 ± 10.1; WC 63.6 ± 12.3; CG 57.7 ± 15.6	T2DM	WHO criteria 2006 Fasting blood glucose levels ≥ 126 mg/dL	Does your mouth usually feel dry?	52.43%/0% WCDM = 47% PCDM = 54%	Yes *p* = 0.0001	Age	4

Sandberg et al. 2001, Sweden [10], and Sandberg et al. 2000, Sweden [9]	102/102 (i) DM: diabetes register in Primary Health Care (ii) CG: Public dental service clinics as the diabetic patients visited for the clinical examination	64.8 ± 8.4/64.9 ± 8.5	T2DM	NS	Patient's subjective feeling of dry mouth	53.5%/28.4%	Yes *p* = 0.0003	Age Gender	5

Chavez et al. 2000, USA [18]	29/23 (i) DM: community-living and geriatric center (ii) CG: geriatric center (iii) Only dentate adults (iv) WCDM: HbA1_c_ ≤ 9% (*n* = 11) (v) PCDM: HbA1_c_ ≥ 9% (*n* = 18)	(i) Mean age NS (ii) Divided into ≤71 years (14/9) and >71 years (15/14)	T2DM	Blood glucose levels ≥ 140 mg/dL at 2 hours after oral glucose tolerance test	Does your mouth frequently feel dry? Does your mouth feel dry when you are eating a meal? Do you have difficulties swallowing foods if you eat without additional fluids?	Data not shown	No	Age Gender Race distribution	2

(2) *Studies in adults NIDDM*									

Zielinski et al. 2002, USA [11]	32/40 (i) DM/CG: Center for Aging at the University Medicine and Dentistry School (ii) Dentate patients with no fewer than 10 teeth present. Patients with a diagnosis of severe dementia and those taking anticoagulants, needing antibiotic prophylaxis or taking antibiotics on the day of examination were excluded.	71 ± 7/74 ± 8	NIDDM	NS	Does your mouth frequently feel dry? Does your mouth feel dry when you are eating a meal? Do you have difficulties swallowing foods if you eat without additional fluids? Response ≥ 2 diagnosis of dry mouth	50%/30%	No *p* = 0.08	Gender Age Diuretics Antidepressants use	3

(3) *Studies in children and adolescents T1DM*									

Javed et al. 2009, Pakistan [12]	48/40 (i) DM: diabetic care unit of a local hospital (ii) CG: oral health centre (iii) Smokers, hepatitis B or C, AIDS, HIV, and narcotic drug used are excluded (iv) WCDM: HbA1_c_ levels < 6.5 (*n* = 12) (v) PCDM: HbA1_c_ levels ≥ 6.5 (*n* = 36)	15 (10–19)/14.6 (10–19)	T1DM	NS	Does your mouth usually feel dry, especially during meals?	WCDM = 80% PCDM = 100% CG = 0%	Yes (DM/CG)	Socioeconomic status	3

DM, diabetes mellitus; WCDM, well controlled diabetes mellitus; PCDM, poorly controlled diabetes mellitus; CG, control group; T1DM, type 1 diabetes mellitus; T2DM, type 2 diabetes mellitus; NIDDM, non-insulin-dependent diabetes mellitus; JBI, Joanna Briggs Institute Prevalence Critical Appraisal Tool.

**Table 3:** Salivary flow rate/hyposalivation studies.

Author, publication year, country	Study population (DM/CG)	Mean age (years) DM/CG	Type of diabetes	DM diagnosis	Type and QFR mL/min	Definition of hyposalivation	Hyposalivation in DM/CG%	Significant association	Matched variables (DM/CG)	JBI scoring
(1) *Studies in adults T1DM*										

Edblad et al. 2001, Sweden [13]	41/41 (i) DM: Department of Paediatrics, Medical Centre Hospital. T1DM since childhood (ii) CG: randomly chosen from the Swedish register (iii) WCDM: HbA1_c_ ≤ 8% (*n* = 26) (iv) PCDM: HbA1_c_ > 8% (*n* = 15)	21 (1.6)/21 (1.6)	T1DM	NS	SWS (paraffin, spitting method) (i) DM: 1.30 (ii) PCDM: 1.31 (iii) WCDM: 1.24 (iv) CG: 1.54	—	—	Nonsignificant (NS)	Age Gender Living in the same county	6

(2) *Studies in adults IDDM*										

Ben-Aryeh et al. 1988, Israel [22]	35/31 (i) DM: Consecutive patients from diabetes service and research unit (ii) CG: healthy volunteers from the hospital staff who were taking no drugs including oral contraceptives	31.2 ± 7.4/29 ± 6.2	IDDM	NS	UWS (spitting method) 0.35 ± 0.24/0.48 ± 0.23	—	—	Yes (*p* = 0.036)	Age Gender	2

(3) *Studies in adults T2DM*										

Lasisi and Fasanmade 2012, Nigeria [15]	20/20 (i) DM: endocrine unit of the medical outpatients department, University College (ii) CG: members of the university community	58.4 ± 10.6/50.2 ± 9.2	T2DM	NS	UWS (spitting method) 0.5/0.75	—	—	Yes (*p* = 0.04)	Gender	3

Vasconcelos et al. 2010, Brazil [7]	40/40 (i) DM: endocrinology service of center for specialized medical care (ii) CG: Stomatology Clinic of School of Dentistry (iii) Smokers, drinkers, pregnant, edentulous, receptors of salivary gland surgery, radiotherapy of the head and neck region, Sjögren syndrome, rheumatoid arthritis, or lupus erythematosus excluded	57.7 ± 8.9/50.2 ± 12.3	T2DM	NS	UWS and SWS (spitting method) (i) UWS: 0.21 ± 0.16/0.33 ± 0.20 (ii) SWS: 0.63 ± 0.43/1.20 ± 0.70	UWS < 0.1 mL/min SWS < 0.5 mL/min	45%/2.5%	Yes (i) UWS (*p* = 0.002) (ii) SWS (*p* = 0.0001) (iii) Hyposalivation (*p* = 0.0001)	Gender Age	3

de Lima et al. 2008, Brazil [16]	30/30 (i) DM/CG: University Dental School (ii) Wearing complete maxillary or maxillary and mandibular dentures.	60 (9)/63 (12)	T2DM	Fasting blood glucose DM ≥ 126 mg/dL	SWS 0.95 (0.61)/1.14 (0.87)	SWS < 0.7 mL/min	NS	Nonsignificant (*p* = 0.331)	Gender Age Race	3

Bernardi et al. 2007, Brazil [8]	82/18 (i) DM: diabetic care unit of a local hospital (ii) CG: oral health center (same city) (iii) Those using total prostheses and mouth breathers were excluded. (iv) WCDM: HbA1_c_≤ 8% (23%) (v) PCDM: HbA1_c_ > 8% (77%)	PC 54.3 ± 10.1; WC 63.6 ± 12.3; CG 57.7 ± 15.6	T2DM	WHO criteria Fasting blood glucose DM ≥ 126 mg/dL CG < 110 mg/dL	SWS (spitting method), (i) PCDM: 0.65 ± 0.62 (ii) WCDM: 0.81 ± 0.47 (iii) CG: 1.95 ± 0.73	—	—	Yes SWS (*p* = 0.001)	Age	4

Dodds et al. 2000, USA [17]	243/240 (i) DM/CG: Participants in the Oral Health San Antonio Longitudinal Study of Aging (ii) CG: those subjects who reported no major health problems and were not taking any medications, other than vitamins or occasional analgesics.	Age is specified by sex per group (i) Female: 61.2 (37–78)/55.3 (37–78) (ii) Male: 63.9 (39–78)/55.9 (36–79)	T2DM	Modified WHO criteria Fasting blood glucose ≥ 126 mg/dL or currently taking diabetic medications	UWS 0.36/0.44 SSP 0.28/036 USS 0.08/0.12 SSS 0.31/0.41	—	—	UWS and USP: nonsignificant; USS and SSS: significantly reduced in DM	NS	5

Chavez et al. 2000, USA [18]	29/23 (i) DM: community-living and geriatric center (ii) CG: geriatric center (iii) Only dentate adults (iv) WCDM: HbA1_c_ ≤ 9% (*n* = 11) (v) PCDM: HbA1_c_ ≥ 9% (*n* = 18)	(i) Mean age NS (ii) Divided into ≤ 71 years (14/9) and > 71 years (15/14)	T2DM	Blood glucose levels ≥ 140 g/dL at 2 hours after oral glucose tolerance test	DM/CG/WCDM/PCDM UWS (spitting method) 0.26 ± 0.29/0.16 ± 0.21/0.14 ± 0.13/0.17 ± 0.25 USP 0.04 ± 0.04/0.04 ± 0.04/0.03 ± 0.02/0.04 ± 0.05 SSP 0.31 ± 0.25/0.21 ± 0.17/0.29 ± 0.19/0.16 ± 0.15	—	—	Nonsignificant (DM/CG) Nonsignificant (CG/WCDM/PCDM)	Age Gender Race	2

(4) *Studies in children and adolescents T1DM*										

Alves et al. 2012, Brazil [19]	51/51 (i) DM: paediatric endocrinology service of hospital (ii) CG: NS glycemic control was established by the determination of glycated haemoglobin concentration	11.3 ± 3.4/11.9 ± 3.4	T1DM	American Diabetes Association criteria (2010)	UWS (spitting method) 0.26 ± 0.14/0.41 ± 0.28	UWS < 0.3 mL/min	NS	Yes UWS (*p* = 0.02)	Socioeconomic status Lived in the same area	2

Javed et al. 2009, Pakistan [12]	48/40 (i) DM: diabetic care unit of a local hospital (ii) CG: oral health centre (iii) Smokers, hepatitis B or C, AIDS, HIV, and narcotic drug used are excluded (iv) WCDM: HbA1_c_ levels < 6.5 (*n* = 12) (v) PCDM: HbA1_c_ levels ≥ 6.5 (*n* = 36)	15 (10–19)/14.6 (10–19)	T1DM	NS	UWS (spitting method) (i) DM: 0.2 (0.1–0.4) mL/min (ii) WCDM: 0.2 (0.1–0.4) mL/min (iii) PCDM: 0.1 (0.1–0.3) mL/min (iv) GC: 0.5 (0.3–0.7) mL/min	—	—	DM/CG, yes (UWS *p* = 0.01) WCDM/PCDM, nonsignificant	Socioeconomic status	3

(5) *Studies in children and adolescents IDDM*										

López et al. 2003, Argentina [20]	20/21 (i) DM: hospital endocrinology service (ii) CG: NS (iii) CG: absence of active disease, no history of drug treatment or therapy within the previous months, and no history of diabetes	9.4 ± 3.9/8.3 ± 1.8	IDDM	NS	UWS = saliva 5 min production collected with sterile syringe No stimulation or spitting 0.15 ± 0.11/0.25 ± 0.13	—	—	Yes (NS)	Gender Socioeconomic status Tanner publeral state between I and III	1

Belazi et al. 1998, Greece [21]	10/10 (i) DM: newly diagnosed diabetic children, Diabetic Department of Paediatric Clinic University Hospital (ii) CG: NS (iii) DM/CG: free from any other acute or systemic disease	6.8 (4–15)/10.5 (5–17)	IDDM	NS	UWS (spitting method), 0.79 ± 0.46/1.06 ± 0.37	NS	—	Nonsignificant (*p* = 0.17)	NS	1

DM, diabetes mellitus; CG, control group; QFR, quantity of flow rate; NS, nonspecific; WC, well controlled; PC, poorly controlled; UWS, nonstimulated salivary flow; SWS, stimulated salivary flow; USP, nonstimulated parotid flow; SSP, stimulated parotid flow; USS, nonstimulated submandibular/sublingual flow; SSS, stimulated submandibular/sublingual flow; JBI, Joanna Briggs Institute Prevalence Critical Appraisal Tool.

## Appendix

### List of Excluded Studies and Reason of Exclusion

[1] F. Javed, HB. Ahmed, A. Mehmood, A. Saeed, K. Al-Hezaimi, and LP. Samaranayake, “Association between glycemic status and oral Candida carriage in patients with prediabetes”,* Oral surgery, oral medicine, oral pathology and oral radiology*, vol. 117, no. 1, pp. 53201358, 2014. (The outcomes were not present.)

[2] E. de la Rosa-Garcia, M. Miramontes-Zapata, LO. Sanchez-Vargas, and A. Mondragon-Padilla, “Oral colonisation and infection by Candida sp. in diabetic and non-diabetic patients with chronic kidney disease on dialysis”,* Nefrologia*, vol. 33, no. 6, pp. 764–770, 2013. (Study about oral candidiasis in DM and non-DM patients.)

[3] DH. Han, MS. Kim, HS. Shin, KP. Park, and HD. Kim, “Association between periodontitis and salivary nitric oxide metabolites among community elderly Koreans”,* Journal of Periodontology*, vol. 84, no. 6, pp. 776–784, 2013. (Study not performed in DM patients. Study about periodontitis.)

[4] G. Teratani, S. Awano, I. Soh, A. Yoshida, N. Kinoshita, T. Hamasaki et al., “Oral health in patients on haemodialysis for diabetic nephropathy and chronic glomerulonephritis”,* Clinical oral investigations*, vol. 17, no. 2, pp. 483–489, 2013. (Study about oral health in haemodialysis patients.)

[5] M. Vesterinen, H. Ruokonen, J. Furuholm, E. Honkanen, and JH. Meurman, “Clinical questionnaire study of oral health care and symptoms in diabetic vs. non-diabetic predialysis chronic kidney disease patients”,* Clinical oral investigations*, vol. 16, no. 2, pp. 559–563, 2012. (Kidney disease patients.)

[6] P. Vestergaard, K. Schwartz, L. Rejnmark, L. Mosekilde, and EM. Pinholt, “Oral bisphosphonate use increases the risk for inflammatory jaw disease: a cohort study”,* Journal of oral and maxillofacial surgery*, vol. 70, no. 4, pp. 821–829, 2012. (This study does not evaluate the outcomes. No DM patients.)

[7] J. Fricton, DB. Rindal, W. Rush, T. Flottemesch, G. Vazquez, MJ. Thoele et al., “The effect of electronic health records on the use of clinical care guidelines for patients with medically complex conditions”,* Journal of the American Dental Association*, vol. 142, no. 10, pp. 1133–1142, 2011. (This study does not evaluate the outcomes. No DM patients.)

[8] AMH. Syrjala, L. Raatikainen, K. Komulainen, M. Knuuttila, P. Ruoppi, S. Hartikainen et al., “Salivary flow rate and periodontal infection - a study among subjects aged 75 years or older”,* Oral diseases*, vol. 17, no. 4, pp. 387–392, 2011. (No DM patients.)

[9] AMH. Syrjala, P. Ylostalo, S. Hartikainen, R. Sulkava, and M. Knuuttila, “Number of teeth and selected cardiovascular risk factors among elderly people”,* Gerodontology*, vol. 27, no. 3, pp. 189–192, 2010. (No DM patients.)

[10] E. de la Rosa Garcia, A. Mondragon Padilla, S. Aranda Romo, and MA. Bustamante Ramirez, “Oral mucosa symptoms, signs and lesions, in end-stage renal disease and non-end-stage renal disease diabetic patients”,* Medicina oral, patologia oral y cirugia bucal*, vol. 11, no. 6, pp. E467–473, 2006. (End-stage renal disease DM patients.)

[11] JM. Sung, SC. Kuo, HR. Guo, SF. Chuang, SY. Lee, and JJ. Huang, “The role of oral dryness in interdialytic weight gain by diabetic and non-diabetic haemodialysis patients”,* Nephrology, dialysis, transplantation*, vol. 21, no. 9, pp. 2521–2528, 2006. (DM and non-DM haemodialysis patients.)

[12] AA. Alavi, E. Amirhakimi, and B. Karami, “The prevalence of dental caries in 5 - 18-year-old insulin-dependent diabetics of Fars Province, southern Iran”,* Archives of Iranian medicine*, vol. 9, no. 3, pp. 254–260, 2006. (Does not evaluate outcomes.)

[13] HW. Boyce, and MR. Bakheet, “Sialorrhea: a review of a vexing, often unrecognized sign of oropharyngeal and esophageal disease”,* Journal of clinical gastroenterology,* vol. 39, no. 2, pp. 89–97, 2005. (Does not evaluate outcomes.)

[14] GE. Sandberg, and KF. Wikblad, “Oral health and health-related quality of life in type 2 diabetic patients and non-diabetic controls”,* Acta odontologica Scandinavica*, vol. 61, no. 3, pp. 141–148, 2003. (Does not evaluate outcomes.)

[15] JS. Mattson JS, and DR. Cerutis, “Diabetes mellitus: a review of the literature and dental implications”,* Compendium of continuing education in dentistry,* vol. 22, no. 9, pp. 757–760, 2001. (Review of the literature.)

[16] CH. Kao, SC. Tsai, and SS. Sun, “Scintigraphic evidence of poor salivary function in type 2 diabetes”,* Diabetes care*, vol. 24, no. 5, pp. 952-953, 2001. (Does not evaluate outcomes.)

[17] EM. Chavez, LN. Borrell, GW. Taylor, and JA. Ship, “A longitudinal analysis of salivary flow in control subjects and older adults with type 2 diabetes”,* Oral surgery, oral medicine, oral pathology, oral radiology, and endodontics*, vol. 91, no. 2, pp. 166–173, 2001. (Longitudinal study.)

[18] AL. Mason, L. Xu, L. Guo, and RF. Garry, “Retroviruses in autoimmune liver disease: genetic or environmental agents?”,* Archivum immunologiae et therapiae experimentalis*, vol. 47, no. 5, pp. 289–297, 1999. (Does not evaluate outcomes. No DM patients.)

[19] RE. Persson, GR. Persson, HA. Kiyak, and LV. Powell, “Oral health and medical status in dentate low-income older persons”,* Special care in dentistry*, vol. 18, no. 2, pp. 70–77, 1998. (Does not evaluate outcomes.)

[20] CP. Robinson, S. Yamachika, CE. Alford, C. Cooper, EL. Pichardo, N. Shah et al., “Elevated levels of cysteine protease activity in saliva and salivary glands of the nonobese diabetic (NOD) mouse model for Sjogren syndrome”,* Proceedings of the National Academy of Sciences of the United States of America*, vol. 94, no. 11, pp. 5767–5771, 1997. (Animal study. Does not evaluate outcomes.)

[21] MR. Quirino, EG. Birman, and CR. Paula, “Oral manifestations of diabetes mellitus in controlled and uncontrolled patients”,* Brazilian dental* journal, vol. 6, no. 2, pp. 131–136, 1995. (Does not evaluate outcomes.)

[22] K. Kimura, K. Ezoe, H. Yokozeki, I. Katayama, and K. Nishioka, “Elevated serum CA125 in progressive systemic sclerosis with pleural effusion”,* The Journal of dermatology*, vol. 22, no. 1, pp. 28–31, 1995. (Does not evaluate outcomes. No DM patients.)

[23] P. Collin, T. Reunala, E. Pukkala, P. Laippala, O. Keyrilainen, and A. Pasternack, “Coeliac disease–associated disorders and survival”,* Gut*, vol. 35, no. 9, pp. 1215–1218, 1994. (Does not evaluate outcomes. No DM patients.)

[24] M. Mogi, M. Harada, T. Kage, T. Chino, and K. Yoshitake, “Two-dimensional electrophoresis of human salivary proteins from patients with sialoadenopathy”,* Archives of oral biology*, vol. 38, no. 12, pp. 1135–1139, 1993. (Does not evaluate outcomes. No DM patients.)

[25] LM. Sreebny, A. Yu, A. Green, and A. Valdini, “Xerostomia in diabetes mellitus”,* Diabetes care*, vol. 15, no. 7, pp. 900–904, 1992. (Type of DM not specified.)

[26] L. Tabak, ID. Mandel, D. Karlan, and H. Baurmash, “Alterations in lactoferrin in salivary gland disease”,* Journal of dental research*, vol. 57, no. 1, pp. 43–47, 1978. (Does not evaluate outcomes. No DM patients.)

[27] S. Conner, B. Iranpour, and J. Mills, “Alteration in parotid salivary flow in diabetes mellitus”,* Oral surgery, oral medicine, and oral pathology*, vol. 30, no.1, pp. 55–59, 1970. (No control group.)

[28] M. Tremblay, D. Brisson, and D. Gaudet, “Association between salivary pH and metabolic syndrome in women: a cross-sectional study”,* BMC oral health*, vol. 12, pp. 40, 2012. (Does not evaluate outcomes. No DM patients.)

[29] AT. Eltas, M. Keles, and V. Canakci, “Assessment of oral health in peritoneal dialysis patients with and without diabetes mellitus”,* Peritoneal dialysis international*, vol. 32, no. 1, pp. 81–85, 2012. (Dialysis patients.)

[30] M. Isola, P. Solinas, E. Proto, M. Cossu, and MS. Lantini, “Reduced statherin reactivity of human submandibular gland in diabetes”,* Oral diseases*, vol. 17, no. 2, pp. 217–220, 2011. (Does not evaluate outcomes.)

[31] P. Gumus, N. Buduneli, S. Cetinkalp, SI. Hawkins, D. Renaud, DF. Kinane DF et al., “Salivary antioxidants in patients with type 1 or 2 diabetes and inflammatory periodontal disease: a case-control study”,* Journal of periodontology*, vol. 80, no.9, pp. 1440–1446, 2009. (Does not evaluate outcomes.)

[32] J. Siudikiene, V. Machiulskiene, B. Nyvad, J. Tenovuo, and I. Nedzelskiene, “Dental caries increments and related factors in children with type 1 diabetes mellitus”,* Caries research*, vol. 42, no. 5, pp. 354–362, 2008. (Longitudinal study.)

[33] S. Aydin, “A comparison of ghrelin, glucose, alpha-amylase and protein levels in saliva from diabetics”,* Journal of biochemistry and molecular biology*, vol. 40, no. 1, pp. 29–35, 2007. (Does not evaluate outcomes.)

[34] KM. Karjalainen, ML. Knuuttila, and ML. Kaar, “Salivary factors in children and adolescents with insulin-dependent diabetes mellitus”,* Pediatric dentistry*, vol. 18, no. 4, pp. 306–311, 1996. (Control group not present.)

[35] P. Canepari, N. Zerman, and G. Cavalleri, “Lack of correlation between salivary Streptococcus mutans and lactobacilli counts and caries in IDDM children”,* Minerva stomatologica*, vol. 43, no. 11, pp. 501–505, 1994. (Flow rate dependent on caries of DM/non-DM patients.)

[36] M. Sutters, C. Brace, E. Hatfield, A. Whitehurst, SL. Lightman, and WS. Peart, “Control of sodium excretion in patients with cranial diabetes insipidus maintained on desamino- 8-D-arginine vasopressin”,* Clinical science*, vol. 85, no. 5, pp. 599–606, 1993. (Does not evaluate outcomes.)

[37] PJ. Lamey, BM. Fisher, and BM. Frier, “The effects of diabetes and autonomic neuropathy on parotid salivary flow in man”,* Diabetic medicine*, vol. 3, no. 6, pp. 537–540, 1986. (Results in relation to autonomic neuropathy.)

[38] M. May, R. Wette, WB. Hardin, and J. Sullivan, “The use of steroids in Bell's palsy: a prospective controlled study”,* The Laryngoscope*, vol. 86, no. 8, pp. 1111–1122, 1976. (No DM patients.)
